# A guide to value of information methods for prioritising research in health impact modelling

**DOI:** 10.1515/em-2021-0012

**Published:** 2021-11-15

**Authors:** Christopher Jackson, Robert Johnson, Audrey de Nazelle, Rahul Goel, Thiago Hérick de Sá, Marko Tainio, James Woodcock

**Affiliations:** MRC Biostatistics Unit, University of Cambridge, Cambridge, UK; MRC Biostatistics Unit, University of Cambridge, Cambridge, UK; and Imperial College London, London, UK; Imperial College London, London, UK; MRC Epidemiology Unit, University of Cambridge, Cambridge, UK; World Health Organization, Geneva, Switzerland; and Center for Epidemiological Research in Nutrition and Health, University of Sao Paulo; MRC Epidemiology Unit, University of Cambridge, Cambridge, UK; and Finnish Environment Institute, Helsinki, Finland; MRC Epidemiology Unit, University of Cambridge, Cambridge, UK

**Keywords:** air pollution, decision theory, design, sensitivity analysis, uncertainty

## Abstract

Health impact simulation models are used to predict how a proposed policy or scenario will affect population health outcomes. These models represent the typically-complex systems that describe how the scenarios affect exposures to risk factors for disease or injury (e.g. air pollution or physical inactivity), and how these risk factors are related to measures of population health (e.g. expected survival). These models are informed by multiple sources of data, and are subject to multiple sources of uncertainty. We want to describe which sources of uncertainty contribute most to uncertainty about the estimate or decision arising from the model. Furthermore, we want to decide where further research should be focused to obtain further data to reduce this uncertainty, and what form that research might take. This article presents a tutorial in the use of Value of Information methods for uncertainty analysis and research prioritisation in health impact simulation models. These methods are based on Bayesian decision-theoretic principles, and quantify the expected benefits from further information of different kinds. The *expected value of partial perfect information* about a parameter measures sensitivity of a decision or estimate to uncertainty about that parameter. The *expected value of sample information* represents the expected benefit from a specific proposed study to get better information about the parameter. The methods are applicable both to situationswhere the model is used to make a decision between alternative policies, and situations where the model is simply used to estimate a quantity (such as expected gains in survival under a scenario). This paper explains how to calculate and interpret the expected value of information in the context of a simple model describing the health impacts of air pollution from motorised transport. We provide a general-purpose R package and full code to reproduce the example analyses.

## Introduction

Simulation models are often used to calculate public health outcomes under different policies or scenarios where disease risk is modified. Outcomes might be defined, for example, by incidence of a disease, or expected life-years lost from any cause, and risk factors might include physical inactivity or air pollution exposure. Models bring together multiple sources of data and assumptions, describe the mechanisms through which scenarios modify risk factors and risk factors affect outcomes, and calculate one or more estimates of the overall health impact. An example is the “Integrated Transport and Health Impact Model” (ITHIM) series of models (de Sá et al. 2017; Garcia et al. 2021; Jaller et al. 2020; Woodcock, Givoni, and Morgan 2013; Woodcock et al. 2014; Wu et al. 2019) that have been used to examine feasible changes in transport behaviours and policies in different settings. These changes are assumed to affect health through three pathways: physical activity related to active transport (bicycling and walking), exposure to air pollution, and road-traffic injuries. See also, e.g. Mueller et al. (2015) for a review of similar applications.

### General health impact model: estimation and decision problems

The model is represented by a deterministic function **Y =** *f* (***θ***) which maps input parameters ***θ*** to (possibly multiple) health outcomes of interest **Y**. The model might be used to answer two kinds of questions: *estimation* or *decision* problems. In either case, the input parameters are uncertain, and we wish to determine the sensitivity of the estimate or decision to each of the inputs, hence to prioritise further research or data collection.

#### Estimation problems

Suppose the model is used to estimate a quantity of interest. For health impact models, **Y** might be a single outcome*Y*,e.g.representing the expected number of cases of a disease(stroke,say)per year averted if pollution emissions from transport were to halve. Section 2 elaborates on how such a model might be constructed. The estimate might be used to inform transport policy, in which multiple (potentially incommensurable) goals might be relevant e.g.health gains from physical activity,achieving ”VisionZero” for traffic injuries,achieving legal limits on air pollutants, journey times, and equity.

#### Decision problems

The model might also be used to decide the optimal policy or action *a* from a finite set *a* = 1,2,…,*A*. Suppose the decision-maker is willing to convert all relevant outcomes under consideration into a single metric, or two metrics that can be directly traded off. The health outcome **Y** is now a vector of *A* elements including the health benefit *Y_a_* (***θ***) of each policy *a*, and we may additionally have a cost *C_a_* (***θ***) for each policy *a.* Assuming a risk-neutral decision-maker is willing to pay a cost of *λ* to gain one unit of benefit, then the optimal policy *a* is the one that maximises NB_*a*_ (***θ***) = *λY*_*a*_(***θ***) − C_a_ (***θ***) (the *net monetary benefit*) where NB_*a*_ (***θ***) is measured in the same monetary unit as the costs. Equivalently we can maximise the *net health benefit* NB_*a*_(***θ***) = *Y_a_(***θ***) − C_a_(***θ***)/λ,* which is in the same unit as the health benefit *Y_a_.* This is a common procedure used in health economic evaluation (Briggs, Sculpher, and Claxton 2006), where *Y_a_(**θ**)* is typically the expected quality-adjusted life year (QALY) gained per person. The willingness-to-pay *λ* depends on the context, for example, NICE in the United Kingdom (National Institute for Health and Care Excellence 2013) use a value of *λ* of around £20,000–30,000 per quality-adjusted life year to decide whether a policy (e.g. whether the health service should fund a new drug) is cost-effective.

In the health impact example (described more fully in Section 2) suppose we have a policy that is expected to reduce pollution emissions from transport, and we wish to determine whether this is cost-effective with respect to health gains from pollution. We might design the model to compute *Y_2_(**θ**),* the expected number of disease cases averted under the policy, compared to the status quo *Y*_1_ = 0, quantify the cost of the policy *C_2_* (***θ***) (with *C*_1_ = 0), and determine the willingness-to-pay *λ* to avert one case. Hence we could compute the net health benefits NB_2_(***θ***) = *Y_2_* (***θ***) − C_2_(***θ***)/*λ,* NB_1_ = 0, and implement the policy if the number of cases averted *Y_2_* (***θ***) is greater than *C_2_(**θ**)/λ,* equivalently NB_2_(***θ***) > NB_1_.

### Uncertainty in health impact models

The parameters ***θ*** are uncertain, and we assume that estimates are based on the *expectation* of outcomes over ***θ***, E_**θ**_(**Y**(**θ**)), or in decision problems, we choose the action *a* that maximises *E*(NB_*a*_(**8**)). Therefore the estimate or decision may be “wrong”, i.e. different from the true ***θ*** or the decision we would make if we knew the true ***θ***.

We take a Bayesian perspective, that is, uncertainty about the input parameters ***θ*** is represented by a joint probability distribution, representing beliefs under all current information. In health impact models, the information might come from a variety of sources, including analyses of primary data,published information, and judgements. While we do not go into detail on how probability distributions on the model parameters might be derived from the data, we give some examples in later sections, and there are guidelines for the related field of health economic modelling (Briggs et al. 2012). In practice, Monte Carlo simulation is used to produce a sample from the uncertainty distribution of **Y** which is implied by the distributions stated for ***θ***, and *E*(**Y**) is estimated by the mean of this sample.

We wish to learn which of the parameters comprising ***θ*** most influence the uncertainty in the estimate or decision, and therefore prioritise further research or data collection. There is a broad literature on sensitivity analysis in epidemiological models (Greenland 1996; Lash et al. 2014), and mathematical models (Frey and Patil 2002; Oakley and O’Hagan 2004; Saltelli et al. 2008). Most commonly in health impact models (de Sá et al. 2017; Woodcock et al. 2014) uncertainty is explored by one-way sensitivity analysis, in which model outputs are compared with uncertain inputs set to alternative values, potentially after uncertainties have been quantified with probability distributions. While this can give an informal guide to what further research may be beneficial, a more formal framework for research prioritisation is given by Value of Information (VoI) methods.

### Value of information

The expected value of information is the expected benefit from learning the unknowns ***θ*** better, hence producing a more precise estimate or reducing the chance of a wrong decision. The main VoI measures are described briefly here, and full theoretical definitions are given in Section 3.

– The*expected value of partial perfect information* (EVPPI) is the maximum expected benefit from further research on an input parameter. This is a measure of sensitivity of the conclusions to the parameter, though answers a slightly different question from one-way sensitivity analysis. The expected value of perfect information (EVPI) is the expected value of learning *all* parameters, hence eliminating all uncertainty.
– The*expected value of sample information* (EVSI) measures the expected benefits from collecting a specific dataset (e.g. of a specific sample size) to inform an uncertain parameter.

The definition of “benefit” depends on the problem and the outcome definition. VoI methods are based on Bayesian decision-theoretic principles, hence can be used for any problem which can be formulated in terms of a decision under uncertainty. As we now describe, this includes *estimation* problems as well as explicit decisions between policies.

VoI methods are well-established in models that are used to make decisions between policies in health economics (Briggs, Sculpher, and Claxton 2006; Fenwick et al. 2020; Rothery et al. 2020). In decision models, the benefits from further information can be measured in the same unit of health or currency as the NB_*a*_.Hence EVPPI and EVSI measure expected monetary or health gains from further information, which will reduce the chance of making a wrong decision. To use VoI for research prioritisation, if NB_*a*_ is a *per person* net benefit, it should be converted to a *population* net benefit by multiplying by the size of the current and future population expected to be affected by the decision, though in practice this size will be uncertain and need to be subjected to sensitivity analysis (see, e.g. Rothery et al. 2020). Then if the benefits are measured in monetary terms, a proposed study can be judged as cost-effective if the EVSI is greater than the cost of sampling, and we might choose the study sample size with the greatest *expected net benefit of sampling* (ENBS, equal to the EVSI minus the study cost).

VoI methods, however, have not been routinely used in health *impact* models, which are used for both estimation and decision problems. While VoI measures are constructed using decision theory, they can also be used for *estimation* problems, by conceiving the problem as the “decision” of what estimate of an uncertain quantity to report.In this case,the benefits of further information can be quantified by *reductions in variance* of the probability distribution representing uncertainty about the quantity of interest, as described in Section 3. The EVPPI in this context is equivalent to a common measure used for sensitivity analysis in mathematical modelling (Borgonovo et al. 2021; Saltelli et al. 2008).

Yokota and Thompson (2004) discussed the challenges of using VoI in health impact models, that include the difficulty of quantifying all uncertainties and the difficulty of computing VoI measures. Recently-developed computational methods, however, have made it straightforward to calculate VoI in very general situations, including both decision-making (Heath, Manolopoulou, and Baio 2017; Heath et al. 2020) and estimation (Jackson et al. 2019).

### Overview of the rest of the article

This paper gives a practical tutorial in the usage of Value of Information methods for epidemiologists and public health researchers developing health impact simulation models. A general-purpose R package is provided to implement the methods, and full code is provided in the supplementary material to reproduce all the analyses.

In Section 2 we describe a simple health impact model and illustrate sources of uncertainty in it. In Section 3 we describe the theory and definitions of various Value of Information measures for both estimation and decision-making problems, and describe straightforward algorithms that can be used to compute them. In Section 4 we illustrate a VoI calculation for the example model, and extend this example model in Section 5 to handle cases where we want to prioritise the collection of different kinds of evidence, demonstrating the corresponding VoI analyses in novel situations. These situations include estimating the relative value of collecting biased vs. unbiased evidence in the presence of different amounts of bias, the value of information in different settings when estimates are derived from hierarchical modelling of multiple similar settings, and the value of obtaining “imperfect” information from literature when we cannot collect primary data.

While a deliberately simple model is illustrated for tutorial purposes, the concepts and methods of VoI analysis that we describe are identical for more complex models, and the computation does not become substantially harder. The only requirements are that the model can be defined as a deterministic function of inputs, with all sources of uncertainty quantified as probability distributions on those inputs, and that we can obtain a sample from the joint uncertainty distribution of all model inputs and outputs.

## Example health impact model

Suppose we want to estimate of the health impacts of a scenario where the emissions of PM2.5 air pollution from transport are *D* times the current amount. For clarity of presentation, we only consider impacts on one population group, to one health outcome (incidence of stroke), but the ideas of VoI analysis generalise easily to more complex models. We know the “background” concentration of PM2.5 *μ,* which is assumed to represent an average exposure, and the proportion *π* of PM2.5 due to transport. Let us assume a simple linear relationship between change in emissions and change in concentrations and exposures. Then the background concentration of PM2.5 in the scenario, from both transport and other sources, is

$$g_{1}(\mu ,\pi ,D)=\mu (\pi D+1-\pi )$$

Then suppose the dose–response relationship between PM2.5 and the incidence of, for example, stroke is described by a nonlinear function *g*_2_(*x*,**d**), taken from Burnett et al. (2014). This maps the PM2.5 exposure level *x* onto the relative risk of stroke: *g*_2_(*x*, **d**) = 1 + *α*(1 − exp(−*β*(*x* − *τ*)*^γ^*)) for an exposure of *x* compared to a baseline exposure of *τ*, for *x ≥ τ*, with *g*_2_(*x*, **d**) = 1 for *x* < *τ*. The parameters of this function are indicated by the vector **d** = *(α, β, γ*, *τ*).

The health impacts of the scenario can then be quantified as the expected number ofstroke cases averted in the scenario compared to the baseline (supposing *D <* 1 and we expect reductions in pollution). Given a baseline incidence of stroke *I*_0_ (expected number of cases per year), which for simplicity is assumed known in this example, this is

$$Y=f(\theta )=I_{0}-I_{0}g_{2}\left(g_{1}(\mu ,\pi ,D),\text{d}\right)/g_{2}(\mu ,\text{d})$$

where ***θ*** is the vector of all unknown parameters: (*μ*, *π*, **d**) = (*μ*, *π*, *α*, *β*, *γ*, *τ*), and *D* is assumed to be fixed. Thus if risk is reduced, so that *g*_2_(*v*) is smaller under the scenario, then *Y* > 0 cases of stroke are averted per year, hence the scenario is beneficial. Or if risk is increased, *Y* < 0, then the scenario is harmful.

Note that this is a simplified mechanism for the health impacts of air pollution. For example, individual exposures to air pollution will vary widely according to ventilation and time exposed (Tainio et al. 2016) and the proportion *π* will vary with the setting and be informed by multiple sources of data, such as source apportionment studies or dispersion models (Karagulian et al. 2015).

The model could be used for either estimation or decision making. We might have been asked by some authority to *estimate E_**θ**_(Y*), the expected health impacts of a scenario where transport PM2.5 emissions change by a specific amount *D*, before any specific policy to achieve (or avert) this change is considered.

Alternatively, we might use the model to make an explicit *decision* between policies. In the example, suppose that a decision-maker will implement some policy if its health impacts are estimated to be greater than some value deemed practically significant, say, if the expected number of averted stroke cases *E*(*Y*) *>k*,where*k* is chosen by the decision-maker’s judgement to account for the costs or harms of the policy (in practice, the model would also be extended to acknowledge that the policy might not achieve the intended change *D*). As described in Section 1.1, this defines “net benefit” functions for the policy being considered, NB_2_(***θ***) = *f* (***θ***) − *k*, and for an alternative “policy” defined by the status quo NB_1_(***θ***) = 0, and the policy chosen is the one that maximises the expected net benefit.

The parameters are all known with some uncertainty, and we wish to determine the expected value of further research to gain more information about the parameters, hence to improve confidence in the estimate or decision resulting from the model. To use VoI methods for this purpose, the information about the parameters should be described by probability distributions.

### Illustration of model parameter and output uncertainty

Firstly we will define the distributions used for the parameters in the example model. In a later section “Structuring and parameterising health impact models to enable VoI analysis” we describe variants of the model that might be used in situations where the available evidence is different, thus we wish to prioritise the collection of different kinds of data.

**Figure 1:**
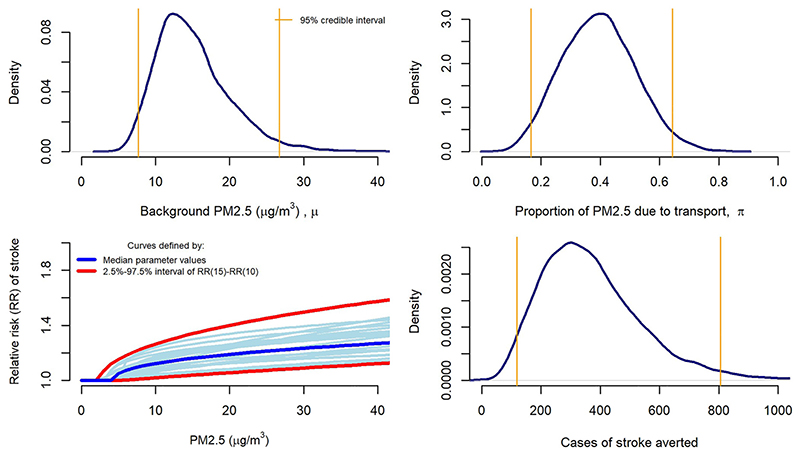
Input and output uncertainty distributions in the example health impact model.

A log-normal distribution, with mean 2.7 and standard deviation 0.3 on the log scale, is assumed for the background PM2.5 concentration *μ,* which ranges from about 10 to 30 *μ*g/m^3^ (Figure 1). A Beta(5.7,8.9) distribution is used for the proportion of PM2.5 *π* due to transport (ranging from around 0.1 to 0.7) and the baseline stroke incidence is fixed at *I*_0_ = 18,530 cases per year. These distributions are based on those used in the ITHIM (Woodcock, Givoni, and Morgan 2013) series of models, which were determined from published estimates from similar contexts and judgements of their relevance. In practice these could varywidely between different settings.

The joint distribution for the parameters **d** = *(α, β*, *γ*, *τ*) relating average PM2.5 exposure to stroke risk was obtained by Burnett et al. (2014) as a random sample as follows. The parameters **d** of curve *g*_2_(*x*, **d**)were estimated from data given by the published relative risks at different exposures *x*. A sample from the joint distribution for **d** was obtained by repeating this estimation with alternative plausible relative risks simulated using the published estimates and their standard errors.

Figure 1 (top row and bottom left) then illustrates the assumed distributions for all of the inputs in the example model. Given these assumptions, the corresponding distribution for the model outputs is obtained by Monte Carlo sampling. For a scenario where transport PM2.5 emissions are *D* = 0.5 times the current amount, the 95% credible interval for averted stroke cases is 118–806 (Figure 1, bottom right). We might then want to know which of the model inputs contribute the most to this uncertainty. A simple way to demonstrate that is by one-way sensitivity analysis. Given alternative values of a parameter, say the 95% credible limits for *μ,* denoted *(μ_L_, μ_U_),* we calculate the resulting values of the model outputs, to indicate the uncertainty in the outputs that is driven by that specific input. Denote the parameters other than *μ* as *v*, so that ***θ*** = (μ, v)is the full set of parameters.

Commonly these resulting outputs are calculated with the *ν* fixed at point estimates $\hat{\nu }$ , as $f(\mu_{\text{L}},\hat{\nu }),\;f(\mu_{\text{U}},\hat{\nu }),$ but this neglects the uncertainty about *v* (McCabe et al. 2020). This uncertainty can be accounted for probabilistically, by calculating the expected model output under these lower and upper limits, *E*(*f*(***θ***)|*μ* = *μ*_L_), *E*(*f*(***θ***)|*μ* = *μ*_U_), where the expected value is calculated with respect to the uncertainty distribution of *v*. This can be computed straightforwardly by Monte Carlo if the distributions of *v* are independent of *μ.* Repeating this procedure for all parameters, and plotting the resulting intervals, gives a *tornado* plot (see, e.g. de Sá et al. 2017).

**Figure 2:**
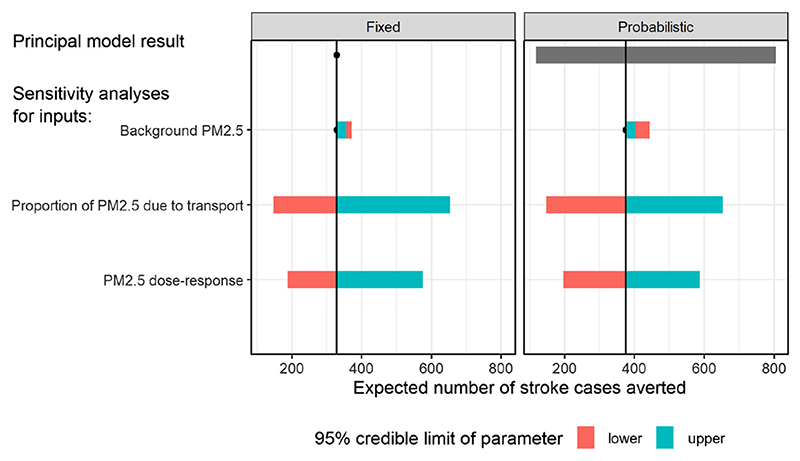
Tornado plot for one-way sensitivity analysis in the example health impact model. Left: non-probabilistic version where input parameters not being varied are fixed at median values. Right: probabilistic version with central estimate defined by the mean output over the distributions of the inputs. The principal results of the models are shown at the top: for the non-probabilistic model this is a point estimate without a credible interval, and for the probabilistic model this is a median and 95% credible interval. The remaining rows illustrate the sensitivity of the model result to variations in the three model inputs.

Tornado plots, using both non-probabilistic and probabilistic methods, are illustrated for the example in Figure 2. In the left panel, parameters not being varied are fixed at point estimates (medians of their distributions) and in the right panel, their uncertainty is accounted for probabilistically. The first row shows the base-case result ofthe model, a simple point estimate under the non-probabilistic model, and an estimate and credible interval under the probabilistic model. The remaining rows show one-way sensitivity analyses: estimates of expected stroke cases averted, contrasted between models that assume different extreme values of particular parameters. The first sensitivity analysis contrasts estimates where the proportion of PM2.5 due to transport is fixed to its lower 95% credible limit of *μ_L_ =* 17%, or to the upper credible limit of *μ_U_ =* 64%. The second sensitivity analysis compares estimates with the background PM2.5 fixed to its lower credible limit of *μ_L_ =* 8, or the upper credible limit of *μ_U_ =* 27.

For the parameters **d** = (*α*, *β*, *γ*, *τ*) governing the dose–response curve *g*_2_(*x*, **d**), instead of varying each parameter separately, they are considered jointly for sensitivity analysis, since their joint distribution is highly correlated, and the ultimate aim is to determine the value of further information about them. Since any data informing dose–response would be likely to inform all four parameters together, varying each parameter independently of the others would not represent the value of further information on dose–response.

Instead, we construct a sensitivity analysis for these parameters by defining two “extreme” instances of the whole curve, which define contrasting strengths of dose–response, hence will produce contrasting estimates when used in a health impact model. To do this, we define the “strength” of dose–response (for a curve with given parameters **d**) as the ratio between the relative risks *ρ* = *g*_2_(*x*_L_, **d**)/*g*_2_(*x*_U_, **d**) for low and high values *x*_L_, *x*_U_ of PM2.5. High and low values are defined, arbitrarily, as average PM2.5 concentrations under the base case and for the scenario with transport emissions halved, which are around *x*_U_ = 15 and *x*_L_ 10 *μ*g/m^3^ respectively. A sample of values is drawn for **d**, which results in a corresponding sample for *ρ*. The joint sample is arranged in order of *ρ*, and the 2.5 and 97.5% quantiles of *ρ* are determined. The two “extreme” dose–response curves are defined by the samples of **d** = *(α, β*, *γ*, *τ*) that produced the 2.5 and 97.5% quantiles of *ρ*. These curves are shown in red in Figure 1 (bottom left). These are used to produce the third row of the tornado plots in Figure 2.

The parameter describing the proportion of PM2.5 attributable to transport appears to have the greatest influence on the health impact of reduced emissions, when varied between its credible limits. The impacts also vary widely between “strong” and “weak” dose responses, while the impact of the background PM2.5 *μ* on the expected stroke cases averted appears to be small and non-monotonic. The final row of the plot (labelled “Overall”) shows the principal result of the analysis, the expected number of stroke cases averted, which is 329 if parameter uncertainty is neglected (left), and 375 when calculated more appropriately as an expected value with respect to parameter uncertainty, with an associated 95% credible interval (bottom right, 120 to 800). Note that if the model is nonlinear, as in this case, neglecting parameter uncertainty and reporting the model output *f (E(**θ**))* evaluated at the parameter estimates gives a biased estimate of the expected model output *E(f (**θ**)),* which is more appropriately obtained as the mean of the Monte Carlo sample.

One-way sensitivity analysis gives an indication of the sensitivity of a model to its inputs, and a decisionmaker will often be interested what the result would be if an uncertain parameter were to take a specific value (see, e.g. McCabe et al. 2020). Implementing it, however, relies on arbitrary choices, particularly if parameters are correlated. Nonlinear relationships between inputs and outputs may also not be detected. One-way sensitivity analysis might be used informally to guide further research, by prioritising research on the parameters whose uncertainty most affects the model output. However, a parameter judged unimportant by one-way sensitivity analysis might sometimes still be valuable to learn better.

A richer and more formal framework for research prioritisation is given by Value of Information analysis, as we introduced in Section 1.3. The expected value of *partial perfect* information (of knowing a parameter exactly) measures sensitivity of a model to an uncertain parameter as the *maximum value of further research* on that parameter. The expected value of *sample* information measures the expected value of a specific study that will give *imperfect* information about one or more uncertain parameters. We now give formal definitions of Value of Information measures, and in Section 4 we explain how they are computed.

## Value of information – theory and definitions

To construct Value of Information measures mathematically, a decision-theoretic perspective is taken, which can handle both decisions between finite actions and estimation problems. Generally, a decision-maker must choose an “action” *a* from a set of actions *A,* to minimise an expected loss *E_**θ**_(L_a_(**θ**)),* with the expected value calculated with respect to the uncertainty distribution of parameters ***θ***. Equivalently this can be framed as maximising an expected net benefit *E*_***θ***_(NB_*a*_(***θ***)), where NB(***θ***) = −*L*(***θ***). In each case, the value of information is defined as the expected loss under current information minus the expected loss given the new information, or equivalently, as the expected net benefit given new information minus the current expected net benefit. We set out how each VoI measure is defined for both estimation and decision-making.

### Models for decisions between finite actions

For decisions among a discrete set of actions, we choose the action that maximises the expected net benefit *E*(NB_*a*_ (**θ**)). In the health impact model example, there are two possible decisions: the proposed action *a* = 2, or the status quo *a* = 1. The expected value of further information is measured as expected gains in net benefit.

#### Expected value of perfect information

The *expected value of perfect information* (EVPI) is the expected net benefit under perfect information minus the expected net benefit under current information. This measures what we would expect to gain (in terms of the outcomes described by the net benefit) if we *learnt all the parameters exactly*. It gives an upper bound to the expected gains from new information. For problems of deciding among a finite set of policies, the decision under current information is the one that maximises the expected net benefit, a* = argmax_*a*_ *E*_***θ***_(NB_*a*_(***θ***)). If we knew ***θ***, then the optimal decision would be *a(**θ**) =* argmax_*a*_NB_*a*_(***θ***). The EVPI is then

(1) $$\text{EVPI}=E_{\theta }{\text{NB}}_{a(\theta )}(\theta )-\underset{a}{\max}\left\{E_{\theta }\left[{\text{NB}}_{a}(\theta )\right]\right\}$$

In the example, a policy is implemented if the expected number of stroke cases averted through that policy is greater than 500. As explained in the Introduction, this implies a net benefit function NB_*a*=2_(***θ***) = *Y*_*a*=2_(***θ***) − 500 for implementing the policy, and NB_*a*=1_(***θ***) = 0 for the “status quo” of taking no action. The optimal action *a* is the one that maximises the expected net benefit *E*(NB_*a*_ (***θ***)) given current knowledge about ***θ***.

An intuitive interpretation of EVPI comes from observing that information only has value if it *changes the decision.* To illustrate this, the EVPI can be interpreted as the expected value (over ***θ***) of the “opportunity benefit” from new information, which is NB_opt_(***θ***)–NB_curr_(***θ***), where *curr* is the decision under current information and *opt* is the optimal decision, that is the decision we would make given perfect knowledge of ***θ***. The “opportunity benefit” can take four different forms, depending on what the current decision under uncertainty is, and whether this will change after learning ***θ***, as shown in the following table.

**Table:** 

*E*(*Y*_2_(*θ*)) (current information)	Current decision (under uncertainty)	*Y_a_* (*θ*)(know *θ*)	Optimal decision(know *θ*)	Opportunity benefit
<500	Status quo	<500	Status quo	0
<500	Status quo	>500	Policy	NB_2_(*θ*) − NB_1_(*θ*)
>500	Policy	<500	Status quo	NB_1_ (*θ*) − NB_2_(*θ*)
>500	Policy	>500	Policy	0

Note that NB_1_ *(**θ**) =* 0. The opportunity benefit is zero if the decision doesn’t change after knowing ***θ***. If new information causes us to adopt the policy, the opportunity benefit is NB_2_ (***θ***) = *Y* (***θ***) − 500, the number of cases averted beyond the minimum that is cost-effective. Or if knowing ***θ*** implies that *Y* (**θ**) 500 and leads us to abandon the policy, we make a cost saving valued in health terms at −NB_2_ (***θ***) = 500 − *Y* (***θ***) cases.

The EVPI is then interpreted as the expected health benefits, either in terms of stroke cases averted, or cost savings on an equivalent scale. This formulation of EVPI also suggests a simple Monte Carlo procedure for computing EVPI, by repeatedly sampling ***θ***, mimicking alternate realities with different values for the true ***θ***, hence estimating the EVPI as the mean of the opportunity benefit over the repeated samples. See Section 4 for more discussion of VoI computation.

#### Expected value of partial perfect information

The *expected value of partial perfect information* (EVPPI) measures what would we expect to gain if we learnt *a specific parameter ϕ* (or group of parameters) exactly, where *ϕ* is a subset of the full vector of parameters ***θ***. For decisions between finite actions, this is

(2) $${\text{EVPPI}}_{\phi }=E_{\phi }E_{\theta |\phi }\left({\text{NB}}_{a(\phi )}(\theta )\right)-\underset{a}{\max}\left\{E_{\theta }\left({\text{NB}}_{a}(\theta )\right)\right\}$$

the expected net benefit of the decision *a (ϕ)* we would make if we knew *ϕ* (partial perfect information), minus the expected net benefit of the decision made under current information. If we knew *ϕ,* the optimal decision would maximise *E_θ|ϕ_* (NB_*a*(*ϕ*)_ (***θ***)).

The inner expectation *E_θ|ϕ_* is taken over the distribution of the uncertain parameters that remain once we have learnt *ϕ,* and since we do not actually know *ϕ* when calculating the EVPPI, we also need an outer expectation *E_ϕ_* to average over the uncertainty about *ϕ.* Computation of the EVPPI (and EVSI) is more challenging than for EVPI, and is discussed in Section 4.

#### Expected value of sample information

The *expected value of sample information* (EVSI) is what we would expect to gain from a study of a particular design and sample size that informs some of the model parameters. Denote by *Z* the data we would obtain from this study, then for finite decision problems,

$$\text{EVS}{\text{I}}_{Z}=E_{Z}E_{\theta |Z}\left({\text{NB}}_{a(Z)}(\theta )\right)-\underset{a}{\max}\left\{E_{\theta }\left({\text{NB}}_{a}(\theta )\right)\right\}$$

the expected net benefit of the decision *a* (*Z*) we would make after the study data are observed and the distribution of the parameters ***θ*** is updated to a posterior ***θ*** |*Z*, minus the expected net benefit of the decision made under current information.

Note that this is computed before *Z* is actually observed. Thus it requires us to define a statistical model that we assume to generate *Z*, which is defined by the parameters ***θ*** that we wish to learn about from the proposed study. In practice, if we can simulate data from this statistical model, and estimate the parameters of interest from the simulated data, we are able to compute the EVSI, as we will explain in Section 4.

The *expected net benefit of sampling* for a proposed study trades off the expected benefits of further research with the costs. It is defined as ENBS_*Z*_ = EVSI_*Z*_ − *C_Z_*, where *C_Z_* are the expected costs of sampling *Z*.

### Value of information definitions in models for estimation

Estimation problems can be expressed in terms of decision theory. The “action” *a* is the choice of point estimate *Ŷ* for a quantity of interest, typically defined as a function of uncertain parameters, *Y = f (**θ**),* and the set *A* of alternative actions is the “support” or set of possible values for *Y*. The “decision problem” is framed in terms of minimising an expected loss, rather than maximising an expected net benefit. The loss function is defined to represent a preference for estimates of *Y* closer to the truth. Here, we use the squared error function *L(Ŷ, Ŷ*) = (*Ŷ* − *Y*)^2^. By minimising a quadratic equation, it can be shown that the “decision” that minimises the expected squared loss is *Ŷ = E_**θ**_* (*Y*), the mean of the belief distribution of *Y*, and the expected loss under the optimal decision is the *variance* of the belief distribution *var* (*Y*) = *E*[(*Ŷ* − *Y*)^2^]. (Note that with an absolute error loss function, the point estimate would be the median of *Y*, and the expected loss would be the mean absolute deviation.)

Since the expected value offurther information equals the expected loss under current information minus the expected loss under the further information, the VoI is expressed in terms of *reductions in variance* of the quantity of interest given better knowledge. Hence the EVPI for estimation problems with squared error loss is simply the variance of the uncertainty distribution of *Y* – an upper bound on any reductions in variance we could achieve by further research to learn ***θ***.

A more useful measure for sensitivity analysis is the EVPPI, which for estimation problems is the expected *reduction in variance* of the model output from perfect information about a particular uncertain parameter *ϕ,*

$${\text{EVPPI}}_{\phi }=\mathrm{var}(Y)-E_{\phi }\left[{\mathrm{var}}_{Y|\phi }(Y)\right]$$

Likewise, the expected value of sample information is the expected reduction in the variance of the model output after the study data *Z* is observed

$${\text{EVSI}}_{Z}=\mathrm{var}(Y)-E_{Z}\left[{\mathrm{var}}_{Y|Z}(Y)\right]$$

Obtaining the expected net benefit of sampling for a proposed study requires variance reductions to be converted to monetary values, by determining the willingness-to-pay for a particular reduction in variance. An example is described in Jackson et al. (2019).

## Computing the expected value of information

All of the VoI measures we describe can be computed given only a single Monte Carlo sample of *R* random values from the uncertainty distribution of the parameters ***θ***, denoted (***θ*** ^(1)^, …, ***θ*** ^(R)^), and the corresponding values of the model output *Y = f (**θ**),* denoted *y* ^(1)^ *, … , y* ^(*R*)^, (for estimation problems) or the net benefit NB_*a*_ (**θ**)(for decision problems).

The methods we describe are implemented in the R package voi, available from https://chjackson.github.io/voi/. In this package, the user supplies a data frame inputs describing the sample of ***θ***, with one row per sampled value and one column per parameter. In decision problems, a corresponding data frame outputs is supplied with one row per corresponding sampled value for the model outputs, and one column per action *a*. In estimation problems, outputs is a vector of the sampled estimates *Y*.

For decision problems, the EVPI can be computed as described in Section 3.1.1, by taking the empirical mean of samples of the opportunity benefit *E*(NB_opt_(***θ*** ^(*r*)^)) − NB_curr_(***θ*** ^(*r*)^). In the voi R package, this is implemented in the function evpi, which is invoked as evpi(outputs, inputs). Recall that for estimation problems, the EVPI is the variance, so can trivially be computed as the empirical variance of the sample *f*(***θ*** ^(1)^), … , *f*(***θ*** ^(*R*)^).

The EVPPI and EVSI are more challenging to compute, as they involve an expectation (over the remaining parameters) of an expectation (over the result of the planned data collection). The definition suggests a “brute force” two-level Monte Carlo estimation procedure, however the sample sizes required for accurate estimates from this method are prohibitive in general. For simpler models (Ades, Lu, and Claxton 2004; Madan et al. 2014) in which the model output is a known linear function of particular parameters, then EVPPI (Madan et al. 2014)orEVSI(Ades,Lu, and Claxton 2004) may be estimated with a single-level procedure. However this will not work for the typical model used to estimate health impacts, where many different sources of information are combined in a complex nonlinear function.

We describe a more general computational procedure for both EVPPI and EVSI that works in a general case, and requires only a single Monte Carlo sample from the model parameters and outputs. This is based on nonparametric regression. This was originally proposed by Strong, Oakley, and Brennan (2014) for discrete decision problems. Jackson et al. (2019) described it for a more general class of situations that included estimation and discrete decision problems.

### Computing EVPPI for a single parameter

**Figure 3:**
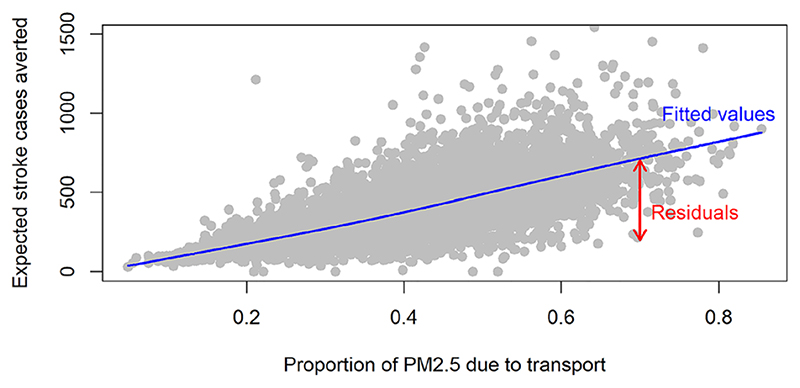
Illustration of using regression to estimate EVPPI as expected reduction in variance of a model output after learning a model input.

The principle is illustrated in the example by plotting the sampled estimates of the expected stroke cases averted, $y^{\left(r\right)}=f(\theta_{1}^{\left(r\right)}),$ against a corresponding sample of values $\theta_{1}^{\left(r\right)}$ from the marginal distribution of one of the parameters ***θ***_1_ that comprise ***θ***, e.g. the proportion *π* of PM2.5 pollution due to transport (Figure 3). For larger values of ***θ***_1_ = π, the health impact *y* ^(*r*)^ is greater, and this relationship can be approximated with a regression model

$$y^{\left(r\right)}=h\left(\theta_{1}^{\left(r\right)}\right)+\epsilon^{\left(r\right)},\;\epsilon^{\left(r\right)}∼N\left(0,\sigma^{2}\right)$$

where *h* () is a sufficiently flexible regression function. While a linear regression provides an adequate estimate of the mean outcome given the predictor in this case, the relationship between a model input and output will not be linear in general – therefore a nonparametric regression function *h* should be used. In this case, a penalised cubic spline was used (Wood 2017).

The fitted value of the function, *ĥ* (*x*), at a value *x*, estimates *E*(*Y*|*π* = *x*), the expected outcome if the uncertain parameter *π* were known to equal *x*. Conversely, the residuals *y* ^(*r*)^ − *ĥ (x* ^(*r*)^) represent the variability in the outcome that is not explained by *π.* In other words, the residual variance *σ* quantifies the uncertainty about *f (**θ**)* that would remain if we were to learn the value of *π*.

Therefore we can estimate the expected value of partial perfect information for an estimation problem, *var*(*Y*) − *E_π_* [*var*_Y|*π*_ (*Y*)] = *var_π_* [*E*_*Y*|*π*_ (*Y*)] (by the “law of total variance”) in two related ways,

(a) as the empirical variance of the fitted values *ĥ (x* ^(*r*)^), an estimate of *var_π_* [*E*_*Y*|*π*_ (*Y*)], roughly interpreted as the variance “explained by” *π.*
(b) as the variance of the *y* ^(*r*)^ minus the residual variance, an estimate of *var* (*Y*) − *E*_*π*_ [*var*_*Y*|*π*_ (*Y*)], the reduction in variance on learning *π.*

Note that the variances and expectations are with respect to the uncertainty distribution of the unknown parameters, hence they are estimated by the empirical variance or mean over samples generated from this distribution.

As described by Strong, Oakley, and Brennan (2014), a similar procedure works for decision problems. Instead of regressing a single model output *y* ^(*r*)^ on the parameters *ϕ* ^(*r*)^, we regress the net benefit value NB_*a*_ (***θ*** ^(*r*)^) on *ϕ* ^(*r*)^ for each alternative action *a*, which produces a sample of fitted values ${\hat{\text{NB}}}_{a}^{\left(r\right)}$ for each *a*. The mean of *R* the fitted values, $\frac{1}{R}\sum_{r=1}^{R}{\hat{\text{NB}}}_{a}^{\left(r\right)},$ estimates *E_**θ**|*ϕ*_* {NB_*a*_ (**θ**)}, the expected net benefit under decision *a*, given perfect information about *ϕ.* The first term in the EVPPI formula (2) is then estimated first by taking the minimum of this quantity over *a* for each *r* (mimicking the decision taken with perfect information), and then taking the mean of these over samples *r*. The second term in formula (2) is estimated by the minimum over *a* of the means over samples *r* of NB_*a*_(***θ*** ^(*r*)^), which corresponds to the decision taken under current information (on the basis of maximum expected net benefit).

### Computing EVPPI for a group of parameters

The method generalises immediately to estimate the EVPPI corresponding to the value of learning *p* ≥ 2 parameters jointly. A nonparametric regression is used with more than one predictor,

$$y^{\left(r\right)}=h\left(x_{1}^{\left(r\right)},\ldots ,x_{p}^{\left(r\right)}\right)+\epsilon^{\left(r\right)}$$

As *p* increases, the computation becomes more challenging. To form the nonparametric regression function *h* (), various methods have been suggested. Strong, Oakley, and Brennan (2014), in the context of health economic models,found that generalized additive models (commonly based on splines) performed adequately up to about *p* = 4, and for higher *p*, Gaussian process regression performed better. Heath, Manolopoulou, and Baio (2016) devised an efficient Gaussian process method based on spatial modelling principles. We have found multivariate adaptive regression splines (MARS) (Friedman 1991; Milborrow 2011) to be efficient and accurate in many situations, however no method is universally reliable, sensitivity analysis is advised, and this is the subject of ongoing research. We would also recommend a sufficient number of Monte Carlo samples are used so that there is enough “data” to be able to reliably identify the nonparametric regression shape when there are several parameters of interest – over 5000 were required in our example.

These methods are all implemented in the voi R package, which provides a common interface to the generalized additive modelling implementation of Wood (2017), the MARS implementation of Milborrow (2011), and the Gaussian process implementations of Strong, Oakley, and Brennan (2014) and Heath, Manolopoulou, and Baio (2016). Each of these by default chooses a best-fitting regression function through a predictive training procedure. Given data frames of the sampled model inputs and outputs, the joint EVPPI for a pair of parameters named “x1” and “x2” is computed with a single command, e.g.

evppi(outputs,inputs,pars=c(“×1”,“×2”))

for decision models, and a similar function evppivar for estimation models. Additional arguments are available to select and fine-tune the computational method. See the package vignette at https://chjackson.github.io/voi/articles/voi.html for worked examples in both decision and estimation problems.

### Computing EVSI

A nonparametric regression method can also be used to estimate the expected value of sample information for a proposed study of a particular design and sample size that aims to estimate one or more of the uncertain parameters (Strong et al. 2015). As for the EVPPI method, this needs only a single Monte Carlo sample for the parameters and model outputs.

The idea is to generate a sample from the *predictive distribution* of data *Z* arising from the study (this is the “prior predictive distribution” if the current information is considered as a prior). For each parameter replicate ***θ*** ^(*r*)^, we generate *Z* ^(*r*)^ from the assumed sampling distribution of *Z*| ***θ*** ^(*r*)^. We then consider how the data would be analysed. Let *T* (*Z*) be a *summary statistic* of the data, that contains all information that *Z* provides about the parameters ***θ***. For each *r*, the simulated data *Z* ^(*r*)^ are summarised using this statistic. The model outcomes *Y* ^(*r*)^ are then regressed on the sample *T Z* ^(*r*)^ to estimate EVSI, in the same manner as they were regressed on the parameters ***θ*** ^(*r*)^ to estimate EVPPI.

If the study information can be summarised in a single scalar, then we regress on a single predictor defined by samples of this summary statistic,

$$y^{\left(r\right)}=h\left(T\left(Z^{\left(r\right)}\right)\right)+\epsilon^{\left(r\right)}$$

or if the study provides *p* different pieces of summary information, a regression with *p* predictors is used

$$y^{\left(r\right)}=h\left(T{\left(Z^{\left(r\right)}\right)}_{1},\ldots ,T{\left(Z^{\left(r\right)}\right)}_{p}\right)+\epsilon^{\left(r\right)}$$

A simple example is where we wish to learn an uncertain probability *p* of some outcome, and we propose to observe the number *Z* out of *N* individuals who have this outcome. Then for each sample *p* ^(*r*)^ from the uncertainty distribution of *p*, an observation *Z* ^(*r*)^ is generated from a Binomial distribution with probability *p* ^(*r*)^ and denominator *N*. The summary statistic is simply *T* (*Z* ^(*r*)^) = *Z* ^(*r*)^, or equivalently we could use the point estimate of *p*, *T* (*Z* ^(*r*)^) = *Z* ^(*r*)^/*N*.

If a summary statistic is not available in closed form, but we can estimate the parameter or parameters of interest *ϕ* from the data *Z*, e.g. by numerically maximising a likelihood, we could define *T (Z* ^(*r*)^) to be the estimate of *ϕ* produced from the simulated data *Z* ^(*r*)^.

The voi R package implements EVSI estimation in this way, allowing the same nonparametric regression methods as for EVPPI. Again, a single command can be used to invoke the EVSI computation, for example,

evsi(outputs,inputs,study=“binary”,n=c(10,100,1000))

for decision models, and a similar function evsivar for estimation models. outputs and inputs are data frames containing samples of model outputs and parameter values as before. The argument study specifies the design of the proposed study, which is “binary” for the simple Binomial example above, and n is asetof alternative sample sizes to compare the EVSI for. Any study design can be specified by supplying a custom R function to simulate summarised data from the study. Worked examples are given in the package vignette at https://chjackson.github.io/voi/articles/voi.html for both decision and estimation problems.

Some other methods of estimating EVSI have been proposed, see Heath et al. (2020) or Kunst et al. (2020) for a review, though the computation for these is more involved, and work is ongoing to implement them in the voi package.

## Value of information analysis in the example health impact model

First we illustrate the EVPPI describing the expected reduction in variance of the model output *Y* (expected stroke cases averted by halving transport PM2.5 emissions). This can be presented in various ways. Most simply, the proportion of variance we can reduce by learning some parameter *ϕ* could be estimated by EVPPI_*ϕ*_/*var*(*Y*). To interpret the EVPPI on an absolute scale, since a standard deviation (SD) is easier to interpret than a variance, we could compute the square root of EVPPI_*ϕ*_ as an estimate of the uncertainty explained by *ϕ,* or the square root of *var* (*Y*) − EVPPI_*ϕ*_ as an *estimate* of the standard deviation of the output that would remain if we learnt *ϕ* (note this is not quite the same as the *expectation* of the remaining SD).

**Figure 4:**
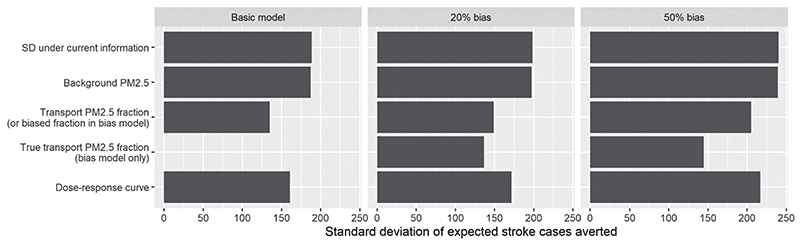
EVPPI analysis in the example health impact model, as predicted standard deviations of expected stroke cases averted given perfect information on each parameter. Basic model (Section 5) and probabilistic bias models with low and high bias (Section 6).

The standard deviation under current information is compared to the estimated standard deviations that would remain after learning the true values of particular parameters in the left panel of Figure 4 for the example (labelled “Basic model”). The greatest reductions in uncertainty are expected from learning the proportion of PM2.5 due to transport, with a more moderate benefit from learning the dose–response function, as was suggested by the probabilistic tornado plot. The single-parameter EVPPIs are estimated with a single penalized cubic spline term, while the joint EVPPI of the four dose–response parameters is estimated with MARS. There is some sensitivity to the nonparametric regression method when estimating the joint EVPPI for a group of parameters, with estimates of the remaining standard deviation, after learning the true values of the four dose–response parameters, varying between 155 and 168.

Now suppose that the model is used for the decision ofwhether to implement a policy which is expected to halve transport PM2.5 emissions, and the policy is adopted ifit is expected to avert 500 or more stroke cases per year. A Monte Carlo sample from the model represents the uncertainty distributions of the number of cases*Y*_2_ averted, and the net health benefit *Y*_2_ − 500. The expected value ofperfect information is calculated from this sample as equivalent to 31 stroke cases averted if we were to learn the exact values of all inputs, hence eliminate the chance of making a wrong policy decision. As when the model was used for estimation, the EVPPI is greatest from learning the fraction *π* of PM2.5 due to transport, at 14 cases, compared to 5 cases from learning the dose–response parameters, or less than 1 case for the background concentration *μ.*

## Structuring and parameterising health impact models to enable VoI analysis

As we have described, to use VoI methods to determine the value of obtaining more information to improve a model,

– a model should be developed with the uncertain quantities represented as parameters, and
– the current knowledge about the parameters should be represented by probability distributions.

In the assessment of health impacts, constructing such a model and distributions may pose a greater challenge than the subsequent computation of the VoI, since the processes being modelled are usually complex and diverse forms of data are required. While this paper does not aim to give a comprehensive guide to modelling and statistical inference for health impacts, we demonstrate, through the worked example, a few principles for uncertainty quantification that may be helpful to structure a health impact model in a way that allows the value of further information to be assessed. The general idea is to use as *flexible a model as possible*.This allows all potential sources of uncertainty to be parameterised, and all potential evidence to be represented, so that we can use VoI methods.

### Describing uncertainty about bias from indirect evidence

Consider *π*, the proportion of PM2.5 attributable to transport. Previously we supposed that there was published information that provides an estimate, with some measure of uncertainty, for this quantity. We assumed we could use this parameter with no further adjustment in the model, since we were confident that this estimate applied directly to our population of interest.

However in practice, we may be less confident in an estimate, either because we know it is based on a different context such as geographical area,time period or population, or because the details of the context are incompletely reported. For example, we may not be confident to apply our estimate of *π* to a different location where industrial sources are expected to contribute a greater proportion of PM2.5 emissions, compared to transport sources. In clinical research, this issue is described as directness or generalisability (Guyatt et al. 2011), for example when generalising results to older patients not included within a clinical trial. This uncertainty could be quantified by extending the model to include a bias parameter.

First we replace the parameter *π* by two different quantities, *π_0_*, the proportion of PM2.5 due to transport in our area of interest, and *π_a_,* representing the average of this quantity over all areas in a region. In the original example, the evidence about *π* was represented by a Beta(5.6, 7.8) distribution. Suppose now we considered this to be biased, and renamed it *π_a_.* We could define the “debiased” context-specific parameter, *π*_0_ = *expit* (logit *(π*_*a*_) + *δ*), then choose the distribution of *δ* to represent our beliefs about the extent of bias. For the above example, we would assume *δ* to be negative, reflecting a lower share of emissions due to transport. If we were unsure of the direction of bias, we might assume *δ* is normally distributed with mean 0 and standard deviation *σ*, which defines a distribution over a valid range of probabilities for *π_0_,* since a logit transform is used. Then we would choose *σ* to represent a transparent belief about the extent of potential bias (in either direction). For example, if we were 90% sure that the target estimate was no more than 20% larger than the published estimate, then we could determine *σ* such that the upper 90% quantile of *π_0_/π_a_* is 1.2 (this can be done numerically, and an R function is provided for this in the accompanying code). Expert elicitation (O’Hagan et al. 2006; Tuomisto et al. 2008) might help to quantify similar kinds of uncertainties.

This is an example of *probabilistic bias modelling*. While there is a wide literature about bias modelling of various kinds (see, e.g. Lash et al. 2014) an advantage of a probabilistic approach is that it directly connects to estimating the value of further research using VoI methods. For example, if we could obtain data about the average over areas for the proportion of PM2.5 due to transport, this value of this would be bounded by the EVPPI of *π_a_*. On the other hand, if we could obtain area-specific data, this would have a value of up to the EVPPI of *π_0_.*

The EVPPI analysis under a probabilistic bias model is illustrated in the example in Figure 4. In the middle plot, we are 90% sure there is no more than 20% bias. In the right hand plot, this probable extent of bias is increased from 20 to 50%. The standard deviation of the outcome under current information increases with the extent of bias. However, the expected *improvements* in precision given further data on the true fraction *π_0_* in the target setting, relative to the current uncertainty, increases with the extent of bias, while the relative value of biased information (parameter *π_a_)* goes down.

### Hierarchical modelling

More generally, if there were estimates for a parameter from multiple similar contexts, such as areas or time periods, then variations between them might be represented with a hierarchical (or multilevel) model. Consider the situation where we do not have an estimate of the proportion of PM2.5 concentrations due to transport for the target area *π_0_,* but we have estimates of the same quantity for *M* other areas. We could estimate the target *π_0_* using a meta-analysis or hierarchical model of the published data (in a similar way to, e.g.Heydari et al. 2020) followed by using VoI methods to estimate the value of further research on the target area.

To illustrate with a simple example, we could specify a sampling model for the published point estimates *x_i_*, *i* = 1, … , *M*, given the true value *π_i_* of the quantity in area *i*, e.g. logit $\left(x_{i}\right)∼N\left(\text{logit}\left(\pi_{i}\right),s_{i}^{2}\right)$ , where *s* ^2^ is a measure of uncertainty around the published estimate, that could be derived from a published credible interval(orelicited belief). Then (after conditioning on any area-level predictors)we might model unexplained variations in the true value between areas *i*, as exchangeable random effects, e.g. logit (*π_i_*) ~ *N* (*μ* ^(*m*)^, *σ* ^(*m*)2^) if there were no predictors. Assigning prior distributions to the hyperparameters *μ^(m)^* and *σ* ^(*m*)^ would then define a Bayesian hierarchical model. The evidence about *π_0_* would then be described by the posterior distribution of *π_0_* given the data from all areas. This distribution could then be employed in the health impact model, followed by a VoI calculation to estimate the value of further research about *π_0_.*

**Figure 5:**
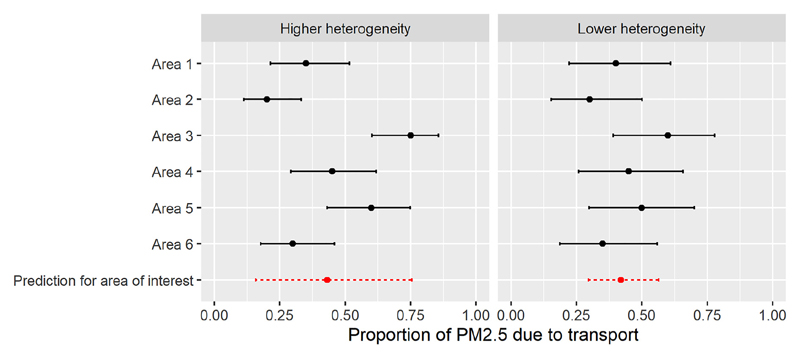
Example health impact model informed by data from multiple areas under a hierarchical model.

Figure 5 shows a hypothetical example, where there are published estimates and credible intervals from six areas. In the left panel, there is large heterogeneity between areas, and in the right panel, there is low heterogeneity. A Bayesian hierarchical model is fitted (using the Stan software, see the supplementary code). The posterior variance of *π_0_* is higher in the left-hand example. In consequence, when this posterior distribution is used in the example health impact model, the standard deviation of stroke cases averted is 669 under high heterogeneity, compared to 247 under low heterogeneity. An EVPPI calculation then predicts the standard deviation after learning perfect information about *π_0_* would be 244 and 103 under high and low heterogeneity respectively. Thus when the variability in the external data is low, and we assume area-level effects are exchangeable, the absolute gains from further information are more modest, since the external data are assumed to be more representative of the required context.

This simple example serves to illustrate the principles of using VoI in the context of a health impact model informed by evidence from a hierarchical model. See, for example, Shaddick et al. (2018) or Heydari et al. (2020) for more sophisticated examples of hierarchical modelling for air pollution exposures.

### Flexible modelling of functional dependencies

Commonly, in health impact models, there are quantities that vary as a continuous function of other quantities. An example here is the “dose–response” curve that describes the relative risk of stroke as a smooth function of the exposure to PM2.5. In other models, there might be quantities that vary as a function of age, or from year to year. The data for particular exposures (or ages, or years) might be weak, thus we aim to “borrow strength” from adjacent data via the fitted curve, while avoiding excessive extrapolation.

We may then want to determine whether additional data on the weakly-informed part of the curve is expected to improve the estimate or decision from the model. For a Value of Information analysis, a functional form should be used which is flexible enough to represent the range of plausible curves given both current data and prior judgements about the curve’s true form. This might be informed by substantive beliefs about the mechanism, as in the dose–response function we illustrated from Burnett et al. (2014). Splines (Wood 2017) or fractional polynomials (Royston and Sauerbrei 2008) can also represent arbitrarily flexible functional relationships.

Once a flexible curve has been defined and fitted to current data, the joint EVPPI for all parameters describing the curve represents the maximum value of further exposure-response data of any kind, as illustrated in the example. Though this assumes that the current data are representative. If additional biases are expected, then these might be introduced as extra parameters through a probabilistic bias model.

### Using EVSI to estimate the expected value of imperfect published information

Once we have determined the *maximum* value of further data informing a particular uncertain parameter, it might then be of interest to determine the expected value of a study of a specific design. In the example, source apportionment studies might give information on the proportion of pollution attributable to transport, and the dose–response curve might be informed by cohort studies ofindividuals exposed to particular concentrations of pollution. In principle, if we can define a statistical model that is assumed to generate the data, in terms of our unknown parameters, we can estimate the expected value of sample information (EVSI).

Designing a realistic primary data collection exercise in this example would involve a complicated extension to the model. However, we can still illustrate some of the principles by calculating the expected value of *imperfect information* that comes with a particular level of uncertainty. For the transport PM2.5 fraction *π,* we might want to determine the expected benefits of information from a study that achieves a certain standard error *σ* for this estimate. We can use the EVSI to do this, by estimating an “effective sample size” behind such a study. Even though a pollution source apportionment study would not involve observing a sample of individuals, information consisting of an estimated proportion with a standard error is approximately equivalent, mathematically, to the information provided by observing a binary outcome from some set of individuals.

This equivalence could be drawn by interpreting the standard error as the standard deviation of the Beta(α,b) posterior that would result from combining a vague Beta(0,0) prior with a “dataset” of *z* eventsout of *n* observations, giving *n = m* (1 − *m*) /*σ* ^2^ − 1, where *m* is the posterior mean *a/ (a* + *b*). Then, estimating *m* by the mean of the current uncertainty distribution of *π,* we can determine *n*, and determine the expected value of a study with standard error *σ* as the EVSI for a study of a binary outcome with *n* observations.

For example, given an estimate of *m =* 0.2 and a standard error of *σ =* 0.1, we would derive *n =* 15, the effective sample size of a Beta(*a* = 15 ×0.2, *b* = 15 ×(1 − 0.2)) posterior distribution. In contrast, a smaller standard error of 0.01, for thesame estimate, would have led to a larger effective sample size of *n* = 1599.

We illustrate this technique in the health impact example by estimating the expected value of a *second* source of information on the proportion of PM2.5 *π* due to transport. The first source of information was representedbyaBeta(5.7,8.9)distribution which has a mean of 0.4and standard deviation of 0.12(see Figure 1). We estimate the expected value of obtaining an additional source of information to supplement the first source, which has the same level of precision as the first source. Using the technique above, we deduce that obtaining an estimate of a proportion with a standard error of 0.12 would be equivalent to performing a study of *n* =42 observations of a binary outcome.

Hence we can use the technique described in Section 4.3 to calculate the expected value of sample information for this second source. For each Monte Carlo sample *π* ^(1)^, *π* ^(2)^, … from the original Beta(5.7,8.9) distribution, we generate a value *Z* ^(*r*)^ from a Binomial distribution with probability *π* ^(*r*)^ and denominator *n* = 42. This represents a potential result of the proposed study, sampled from the predictive distribution of the binary outcome. The “summary statistic” of the study data is simply*T*(*Z*) = *Z*. A regression is then fitted with the sampled model outputs *Y* ^(*r*)^ as the outcome and the set of sampled *Z* ^(*r*)^ as the explanatory variable. The variance of the fitted values from this regression then estimates the expected variance of the model output that would remain after the second source of data is collected (as in Section 4.1). We predict the remaining standard deviation to be 151 for the stroke cases saved after obtaining the second source ofinformation. Thus the information would have a substantial value, in the context of the estimated SD of 135 remaining under perfect information, and the original SD of 188.

## Discussion

Value of Information methods can be used to estimate the expected benefits from further research on uncertain quantities in models that combine different sources of evidence. While they have previously mainly been used in health economic decision models, we have shown how they can be used in health impact models in which there is no explicit policy decision, and the aim of the model is to estimate a quantity of interest. We have demonstrated examples of VoI in a simple model for assessing the health impacts of changes in transport emissions. Such methods might be particularly helpful for prioritising research to inform policy making in contexts with less good data.

As discussed in Section “Illustration of model parameter and output uncertainty”, the EVPPI answers a similar, but not identical, question, to standard one-way sensitivity analysis, predicting gains from further research, rather than comparing model outputs for different inputs. Both methods can be implemented for a low computational cost, thus may complement each other. VoI methods can be used not only as a form of sensitivity analysis (the EVPPI), but also to design specific further research or data collection (the EVSI).

The principles generalise to more complex models. All that is required is to define the outcome or decision of interest, express uncertainties as probability distributions on parameters, and generate a sample from the joint distribution of parameters and outcomes, then the expected value of partial perfect information for a parameter may be calculated as before. Then if we can simulate predicted data from a proposed study of a given design, and summarise the data in a simple form, we can also calculate the expected value of sample information for that study. Sensitivity analysis is recommended if different plausible model parameterisations, or ways of expressing uncertainty, would affect the decision of what further research to do given the VoI estimates, particularly in the context of the costs of further research.

In public health modelling, there are typically many outcomes of interest, that are valued in different ways by different parties. In transport policy, for example, as well as health impacts of pollution, considerations include legal limits on air pollution concentrations, health benefits of physical activity, “net zero” for greenhouse gas emissions, “Vision Zero” for traffic injuries, and travel metrics such as journey times. Therefore there may be several reasonable ways to construct models to estimate outcomes or inform policy decisions, and estimate the value of further information, depending on how the different outcomes are valued. There may also be different forms of the same model applied to different areas, with some parameters shared between the models (e.g. relative risks for a disease given changes in an exposure), and some parameters specific to each area (e.g. pollution concentrations). Learning about the disease relative risks would then be expected to improve the estimates of health impacts aggregated over all areas, while learning area-specific parameters would only improve the estimates for the corresponding area.

Ongoing technical challenges for value of information analysis include computation of the joint EVPPI for a large number of parameters simultaneously (e.g. more than 15), when nonparametric regression becomes more difficult. Also as outlined in Brennan, Chick, and Davies (2006), there are many alternative structures for health policy simulation models, and one of these is “microsimulation”, which works by simulating outcomes for a large population of synthetic individuals, and then taking an average. In these, uncertainty is sometimes not expressed explicitly by placing distributions on inputs, but implicitly, through using a finite sample of observed individual-level data to generate the synthetic population (see Mytton et al. 2018 for an example). Calculating the value of information in this context would be challenging – though could be done in theory by defining a parametric model from which synthetic individuals could be sampled.

While more focused guidance for specific situations would help to make VoI methods more useful in practice, this article explains all the essential ideas necessary to use them in the context of models for assessing health impacts.

## Supplementary Material

The online version of this article offers supplementary material (https://doi.org/10.1515/em-2021-0012).

1

2
